# The microbiome landscape of oral cancer in young patients

**DOI:** 10.1093/jncics/pkag022

**Published:** 2026-04-21

**Authors:** Laveniya Satgunaseelan, Dario Strbenac, Carsten Palme, Tsu-Hui (Hubert) Low, James Wykes, Tim Manzie, Jonathan R Clark, Jean Y H Yang, Ruta Gupta

**Affiliations:** Department of Tissue Pathology and Diagnostic Oncology, NSW Health Pathology, Royal Prince Alfred Hospital, Sydney, Australia; Faculty of Medicine and Health Sciences, Sydney Medical School, The University of Sydney, Sydney, Australia; School of Mathematics and Statistics, The University of Sydney, Sydney, Australia; Sydney Precision Data Science Centre, The University of Sydney, Sydney, Australia; Department of Head and Neck Surgery, Sydney Head and Neck Cancer Institute, Chris O’Brien Lifehouse, Sydney, Australia; Faculty of Medicine and Health Sciences, Sydney Medical School, The University of Sydney, Sydney, Australia; Department of Head and Neck Surgery, Sydney Head and Neck Cancer Institute, Chris O’Brien Lifehouse, Sydney, Australia; Department of Otolaryngology—Head & Neck Surgery, Faculty of Medicine and Health Sciences, Macquarie University, Sydney, Australia; Faculty of Medicine and Health Sciences, Sydney Medical School, The University of Sydney, Sydney, Australia; Department of Head and Neck Surgery, Sydney Head and Neck Cancer Institute, Chris O’Brien Lifehouse, Sydney, Australia; Faculty of Medicine and Health Sciences, Sydney Medical School, The University of Sydney, Sydney, Australia; Department of Head and Neck Surgery, Sydney Head and Neck Cancer Institute, Chris O’Brien Lifehouse, Sydney, Australia; Faculty of Medicine and Health Sciences, Sydney Medical School, The University of Sydney, Sydney, Australia; Department of Head and Neck Surgery, Sydney Head and Neck Cancer Institute, Chris O’Brien Lifehouse, Sydney, Australia; Royal Prince Alfred Institute of Academic Surgery, Sydney Local Health District, Sydney, Australia; NHMRC Centre of Research Excellence for Applied Innovations in Oral Cancer, Sydney, Australia; School of Mathematics and Statistics, The University of Sydney, Sydney, Australia; Sydney Precision Data Science Centre, The University of Sydney, Sydney, Australia; NHMRC Centre of Research Excellence for Applied Innovations in Oral Cancer, Sydney, Australia; Charles Perkins Centre, The University of Sydney, Sydney, Australia; Department of Tissue Pathology and Diagnostic Oncology, NSW Health Pathology, Royal Prince Alfred Hospital, Sydney, Australia; Faculty of Medicine and Health Sciences, Sydney Medical School, The University of Sydney, Sydney, Australia; Department of Head and Neck Surgery, Sydney Head and Neck Cancer Institute, Chris O’Brien Lifehouse, Sydney, Australia; NHMRC Centre of Research Excellence for Applied Innovations in Oral Cancer, Sydney, Australia

**Keywords:** oral squamous cell carcinoma, microbiome, alpha diversity, beta diversity, early onset cancer

## Abstract

**Background:**

The incidence of oral squamous cell carcinoma (OSCC) is rising in patients under the age of 50, without smoking or alcohol abuse. Viruses are not a causative factor of OSCC in younger patients. The oral microbiome has not been evaluated in this unique patient cohort for a potential bacterial etiology.

**Methods:**

We report the bacterial diversity and composition of the largest cohort of OSCC patients with whole genome sequencing (*n* = 72) and compare it with oral mucosa from healthy controls (*n* = 10) using the Strengthening the Organization and Reporting of Microbiome Studies guidelines.

**Results:**

The microbial diversity between tumor, normal mucosa from cancer patients and healthy control mucosa is significantly different, with specific species (*Streptococcus mitis*, *Haemophilus haemolyticus,* and *Cutibacterium acnes*) reduced in normal mucosa of cancer patients as compared with healthy controls (adjusted *P* < .05). The microbial diversity is significantly higher in younger patients as compared with older patients (*P* < .001), with a reduced abundance of anaerobes in older patients (*Aggregatibacter segnis*, *Gemella morbillorum*, *Peptostreptococcus stomatis*, *Filifactor alocis*, and *Porphyromonas endodontalis*; adjusted *P* < .05).

**Conclusion(s):**

The OSCC tissue of younger patients is significantly more polymicrobial, and their OSCC microbiomes harbor more anaerobic bacteria as compared with older patients. This compositional difference builds the hypothesis that the oral microbiome of younger OSCC patients may have a more hypoxic, immunosuppressive tumor microenvironment with its associated implications for treatment resistance and a potential link to baseline poor dentition.

## Introduction

Oral squamous cell carcinoma (OSCC) represents a major global cancer burden, with a worldwide incidence of 380 000 cases and 180 000 deaths per year.^1^ Oral squamous cell carcinoma has traditionally been a disease of older males with tobacco and alcohol use as key risk factors.^2^ However, with the decline in smoking in developed world, the incidence of oral cancer is rising in young patients with minimal smoking history.^3^ The cause for this rising incidence of OSCC with minimal risk factors is being investigated. Interestingly, comprehensive molecular profiling of the cancer tissues, including whole genome sequencing (WGS), has not identified significant differences in the genomic landscape,^4^^,^^5^ raising the possibility that host factors such as the microbiome of the oral tissues may play a role.

The oral cavity hosts more than 700 microorganisms, including viruses, bacteria, and protozoa.^6^ In contrast to oropharyngeal SCC, where human papillomavirus plays a major carcinogenic role, viruses have not been identified in tongue SCC from young women.^7^ Bacteria have been shown to cause cancer in other organ systems such as *Helicobacter pylori* and gastric cancer.^8^ Numerous studies have used 16S rRNA to identify causative bacteria in OSCC over the last decade.^9-11^ Candidate genera including *Fusobacterium*, *Prevotella*, *Corynebacterium*, and *Porphyromonas*^12^ have been proposed but no single bacteria or a characteristic group of bacteria has consistently been associated with OSCC.^13^

Herein, we present the microbiome analyses of the largest cohort of young, never-smoking patients with OSCC to date, and show key compositional differences between young and older patients. In this study, we have used WGS approaches^6^^,^^13^^,^^14^ to overcome the detection biases associated with 16S rRNA sequencing used in many oral microbiome studies.^9-11^ This study follows the checklist “Strengthening The Organization and Reporting of Microbiome Studies (STORMS),” established by an international multidisciplinary group of microbiome researchers to ensure robustness and reproducibility.^15^ Furthermore, in addition to normal and cancer tissues from OSCC patients, this study also includes WGS analyses of normal oral mucosa from healthy volunteers as controls.

## Methods

The study was conducted in accordance with the ethical standards of the Helsinki Declaration. Institutional human ethics committee approval was obtained from Sydney Local Health District Health Human Research Ethics Committee (X19-0282/ETH12165). Written informed consent was obtained from all patients.

### Study design and tissue collection

Seventy-two patients with fresh frozen tissue samples taken from their OSCC were selected from the Sydney Head and Neck Cancer Institute biobank. A cutoff of 50 years of age was used to classify the study cohort into “young” vs “older” patients. All patients were subject to the same preoperative protocol. Tissue collection occurred under sterile conditions at the time of surgical resection for the OSCC. Three to 5 cubic millimeters of tissue was collected from areas of: (1) macroscopically viable tumor and (2) adjacent macroscopically normal mucosa. In addition, normal oral mucosa was collected from 10 volunteers undergoing surgery for benign lesions. All collected tissue was immediately snap frozen in liquid nitrogen and stored at −80 ºC. In some resections, collection of normal mucosa was not possible due to potential disruption of surgical margins, and therefore blood collected preoperatively was used as a matched normal.

### Nucleic acid extraction, WGS, and data processing

Nucleic acid extraction and WGS were performed as previously published,^4^^,^^5^^,^^7^ using the same cell lysis technique as microbiome studies.^16^ All samples had DNA of sufficient quantity and quality, and available clinicopathological data. The WGS data were processed to extract nonhuman reads and microbial identification and quantification. Sequencing coverage and quality statistics are listed in Table S1.

As a first step, the Burrows-Wheeler Aligner Maximal Exact Matches read aligner version 0.7.17^17^ was used to align reads to the hg38 human reference genome and its alternate contigs. Then, samtools version 1.19^18^ was used with the argument “-f 12” to extract unmapped read pairs.

Microbial identification and quantitation were carried out using Metagenomic Phylogenetic Analysis (MetaPhlAn) version 4.0.6,^19^ a stringent taxonomy-based microbial classifier that uses the detection of species-specific unique marker genes. MetaPhlAn version 4.0.6 was used with default parameters and the accompanying June 2023 version of ChocoPhlAn, a pangenome database to generate the species proportions for all samples. Four hundred and eighty-eight species were identified in at least 1 sample. To assess data reproducibility, 3 cancer samples had 2 technical replicates of WGS and each pair of replicates was subset to the species which had nonzero abundance in at least 1 sample. The concordance was assessed by calculating the Pearson correlation between the replicate pairs (Figure S1). Two out of 3 pairs of replicates had a large positive correlation, indicating that the measurements were generally reproducible, although the negative correlation of patient OSCC_24’s sample pair indicates that this consistency may sometimes be lower than desirable. Independent filtering was applied after assessment of data reproducibility.

#### Clinical covariates

Comparisons were made by tissue type (control mucosa from healthy volunteer, normal mucosa from OSCC patients, OSCC tissue). Comparisons were also made by clinical covariates, including age, sex, self-reported smoking practices, anatomical subsite, recurrence, metastasis, and death from disease. Clinicopathologic covariates were collected from patient medical records.

### Statistical analysis

Diversity analyses were performed on the entire cohort (*n* = 72).

*Alpha diversity*, defined as quantification of the diversity of bacterial species within a single sample (within-sample diversity), was calculated by the Shannon index. To determine each sample’s Shannon index, the alpha function from Bioconductor package microbiome version 1.26 was used with the parameter index = “shannon”.

*Beta diversity*, defined as a quantitative measure of the difference between microbial communities (between-sample diversity) in different patients, was determined using the Bray-Curtis dissimilarity between samples via the vegdist and adonis2 functions from CRAN (Comprehensive R Archive Network) package vegan version 2.6, and Principal Coordinate Analysis (PCoA) ordination was performed by the pcoa function of CRAN package ape version 5.8.^20^

#### Modeling used for bacterial abundance

Analyses for bacterial abundance could be performed on those samples that included tumor and matched normal mucosa (*n* = 51) and the results of the paired cancer-normal linear model are presented for bacterial abundance (see Supplementary Methods and Figure S2). Independent filtering was applied to the species proportional abundance matrix^21^ to retain only those species observed in at least 20 samples, reducing the number of species to perform hypothesis testing from 488 to 53. For hypothesis testing of each species’ proportional abundance and its association to tissue types and clinical conditions, the linda function from CRAN package MicrobiomeStat version 1.2^22^ was employed. LinDA uses a centered log ratio transformation of the data.^23^^,^^24^

#### Indices of treatment failure

Survival data were obtained from a prospectively held database. Based on the outcomes collected, the OSCC patient cohort was grouped into those who suffered events such as recurrence, metastasis, or death within 2 years, and those who did not suffer these events for at least 2 years. Survival analyses were performed using alpha diversity, as has been previously used in pancreatic cancer.^25^^,^^26^

## Results

**Figure 1.**
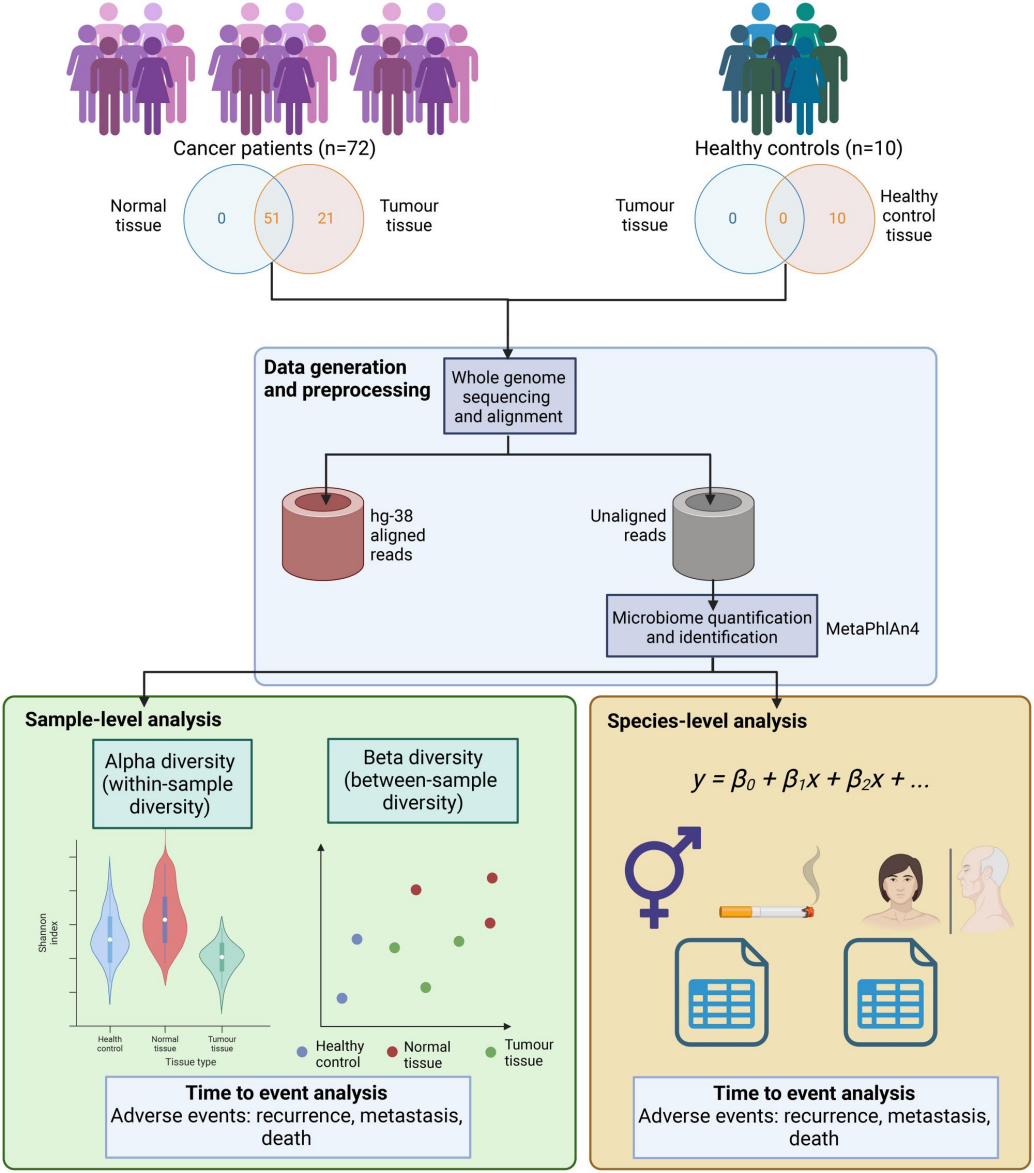
Overview of the study design and the key aspects of the analysis. Flowchart depicting the number of patients in each group and the computational data processing and statistical modeling conducted. Created in https://BioRender.com.

The study cohort comprised 82 individuals, including 72 OSCC patients and 10 healthy volunteers as controls. The OSCC cohort includes 39 patients under the age of 50 years (54%; median age = 41 years) and 33 patients 50 years and over (46%; median age = 77 years). There were 42 males (58%) and 30 females (42%); 52 patients had never smoked (72%) and 20 had smoked tobacco at some time (28%). The demographics are summarized in Table 1. Of the 72 OSCC patients, 51 had paired tumor and normal tissue available for matched analysis (Figure 1). Three patients contributed 2 cancer samples each, from their recurrence or metastases in addition to the primary tumor samples. The remaining patients had matched blood normal, as opposed to normal tissue, and therefore were excluded from the paired analysis. The 10 healthy volunteers providing normal mucosa had never smoked (median age = 48.5 years [range = 36-79 years]; 4 males and 6 females) and remained cancer free. Participant level data are available in Tables S2 and S3.

**Table 1.** Cohort demographics for overall cohort, cohort <50 years and cohort ≥50 years.

Overall cohort (*n* = 72)	No. or median (range)
Age, years, median (range)	50 (19-90)
Sex	
Male	42
Female	30
Smoking status	
Ever smokers	20
Nonsmokers	52
Events	
Recurrence	15
Metastasis	7
Death	10
Cohort <50 years (*n* = 39)	
Age, years, median (range)	41 (19-49)
Sex	
Male	22
Female	17
Smoking status	
Ever smokers	14
Nonsmokers	25
Events	
Recurrence	6
Metastasis	2
Death	5
Cohort ≥50 years (*n* = 33)	
Age, years, median (range)	77 (50-90)
Sex	
Male	20
Female	13
Smoking status	
Ever smokers	6
Nonsmokers	27
Events within 2 years	
Recurrence	9
Metastases	5
Death	5

### The microbial communities in the normal mucosa and cancer tissues are significantly different

**Figure 2.**
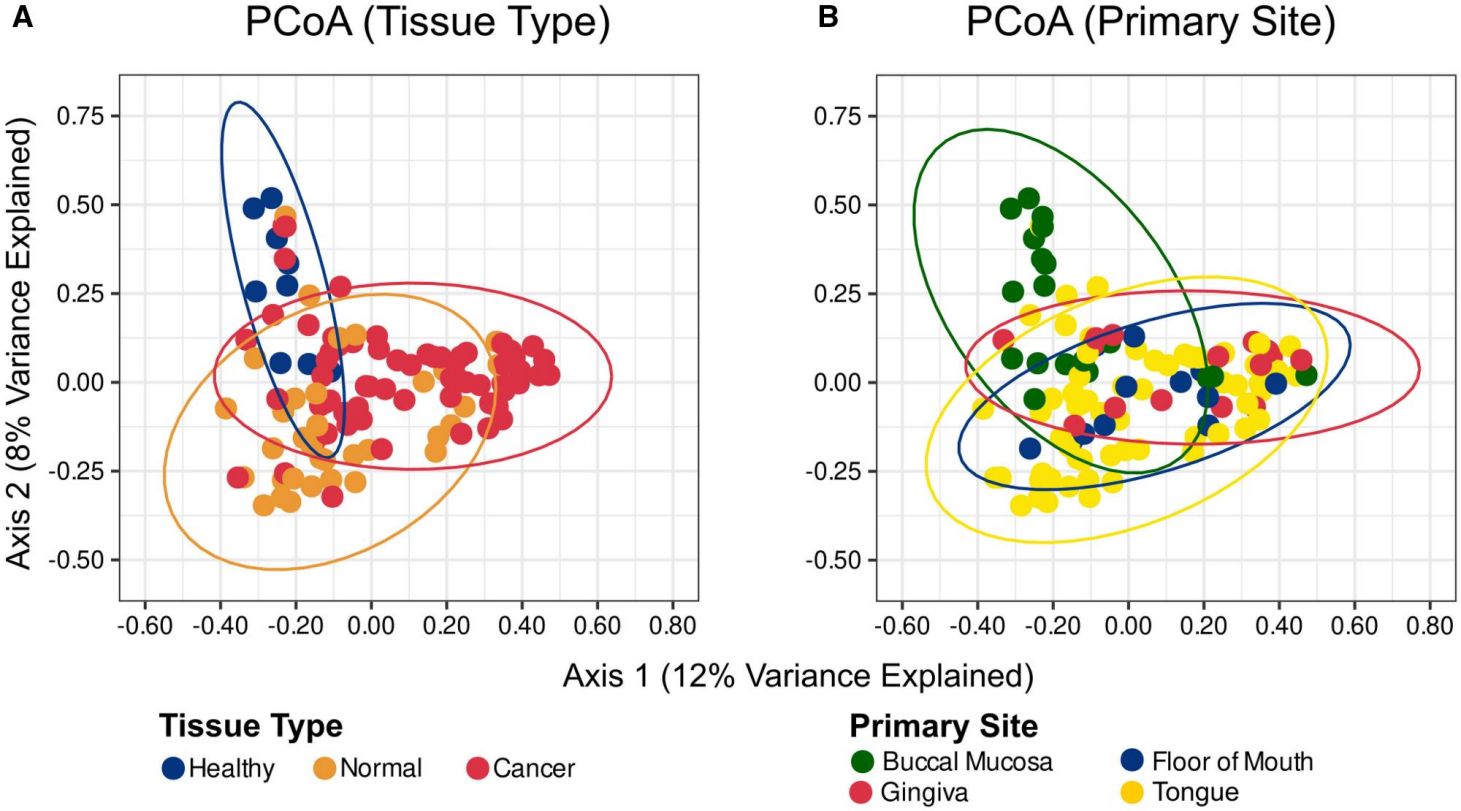
Principal coordinate analysis (PCoA) plot comparing beta diversity between **(A)** tissue types (healthy control tissue vs normal to tumor vs tumor tissue) and **(B)** primary site.

Figure 2A demonstrates that the beta diversity (between-sample diversity) of healthy control mucosa, normal mucosa from OSCC patients, and tumor tissue form distinct clusters (*R*^2^ = 0.07, *P* < .00001). There were well-defined separate clusters of tongue and buccal mucosa subsites based on beta diversity (Figure 2B) and an overall test for differences was statistically significant (*R*^2^ = 0.06, *P* < .00001). Distinct clustering by beta diversity was not observed when clinical co-variates such as age, sex, or smoking status were analyzed (Figure S3).

### Specific oral commensals have reduced abundance in normal mucosa from OSCC patients as compared with control mucosa from healthy volunteers

Three known oral commensals, including anaerobes such as *Streptococcus mitis*, *Haemophilus haemolyticus*, and *Cutibacterium acnes,* were found to be significantly reduced (*P* < .05; Table S4) in normal tissues from OSCC patients as compared with healthy controls.

### Certain oral commensals have increased abundance in OSCC overall but have reduced abundance in older OSCC patients

Numerous oral bacterial species show a significantly higher abundance in tumor compared with normal tissue adjacent to OSCC, including *H. haemolyticus*, *Fusobacterium nucleatum*, *Aggregatibacter segnis*, *Gemella morbillorum*, *Peptostreptococcus stomatis*, *Filifactor alocis*, and *Porphyromonas endodontalis* (*P* < .05; Table S5).

Table 2 shows that the abundance of *A. segnis*, *G. morbillorum*, *P.  stomatis*, *F. alocis*, and *P. endodontalis* was lower in tumor samples from older OSCC patients (*P* < .05; Table 2). No statistically significant differences in bacterial abundance were identified by sex, smoking status, or primary subsite (Figure S4; Figure 2).

**Table 2.** Abundance of bacterial species in tumor samples with matched normal tissue older vs young OSCC patients (adjusted *P* < .05, Benjamini-Hochberg).

Bacterial species	log2 FC	SE	Stats	Adj. *P*
*Aggregatibacter segnis*	−2.4	0.63	−3.9	.012
*Gemella morbillorum*	−3	0.84	−3.6	.012
*Peptostreptococcus stomatis*	−2.3	0.66	−3.4	.012
*GGB1843 SGB2516*	−2.7	0.82	−3.3	.012
*Filifactor alocis*	−2.6	0.79	−3.3	.012
*Alloprevotella sp oral taxon 473*	−2.8	0.85	−3.3	.012
*Porphyromonas endodontalis*	−3.4	1	−3.3	.012
*Catonella SGB69305*	−2.5	0.82	−3.1	.02

Negative log-fold changes indicate the species are higher in young patients. Abbreviations: FC = fold change; Adj. = adjusted

### Younger OSCC patients show greater diversity of microbial species than older OSCC patients

**Figure 3.**
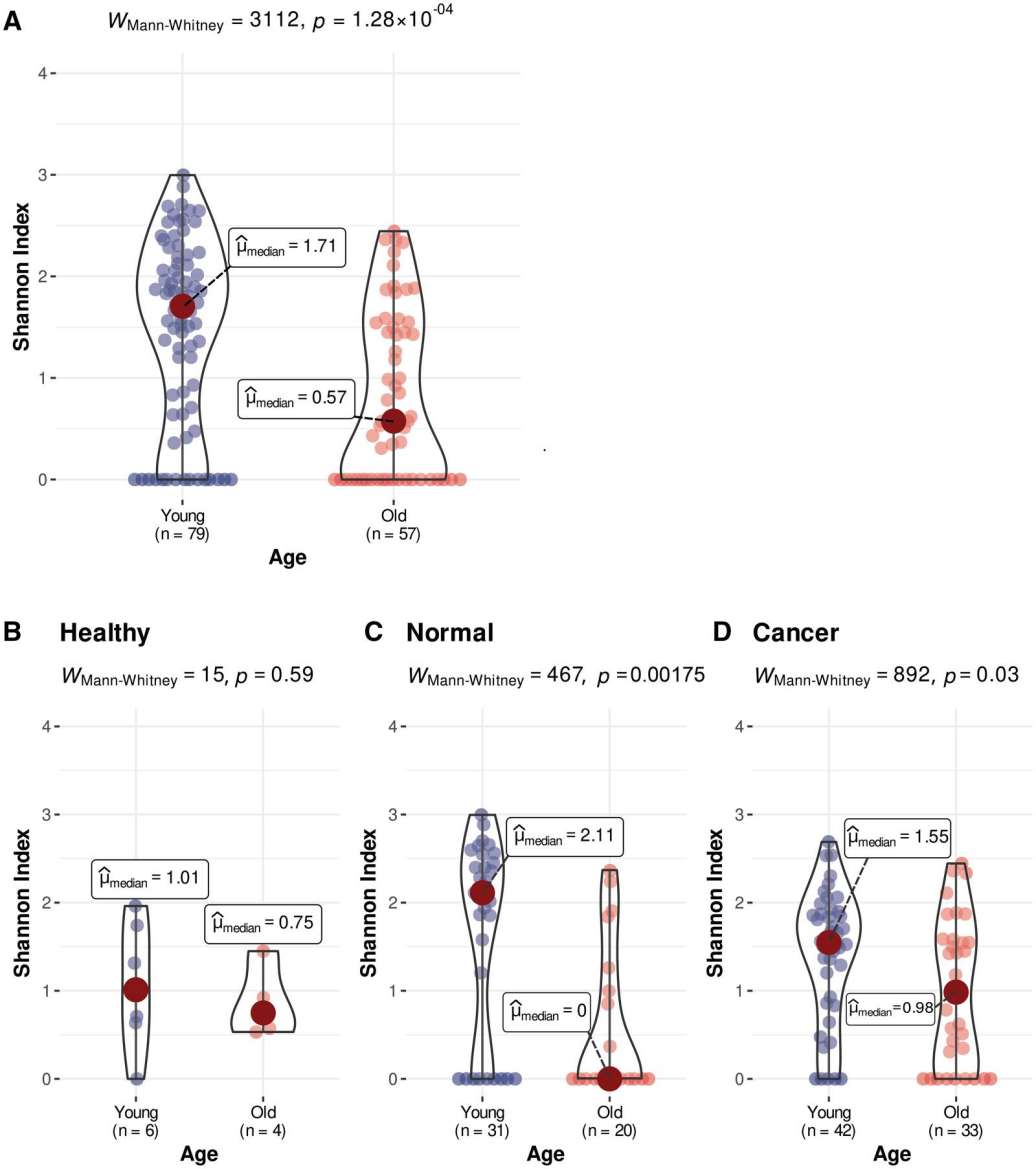
Boxplot comparing the alpha diversity between young (<50 years old) vs old (≥50 years old) for **(A)** all individuals across tissue types; **(B)** healthy controls only; **(C)** normal tissue to tumor; and **(D)** tumor tissue.

Figure 3A shows a statistically significant (*P*≤.001) difference in the alpha diversity between young and older OSCC patients, irrespective of tissue type. The younger OSCC patients have significantly higher alpha diversity, including in normal (Figure 3C, *P* = .002) and tumor tissues (Figure 3D; *P* = .03). However, there was no significant difference (*P* = .59; Figure 3B) in alpha diversity between young and older healthy controls.

No significant difference was found when we examined the alpha diversity between the tissue types or other clinical covariates, including sex, smoking status, or subsite, regardless of tissue type (Figure S5).

### No difference in overall or disease-free survival was observed based on alpha diversity

**Figure 4.**
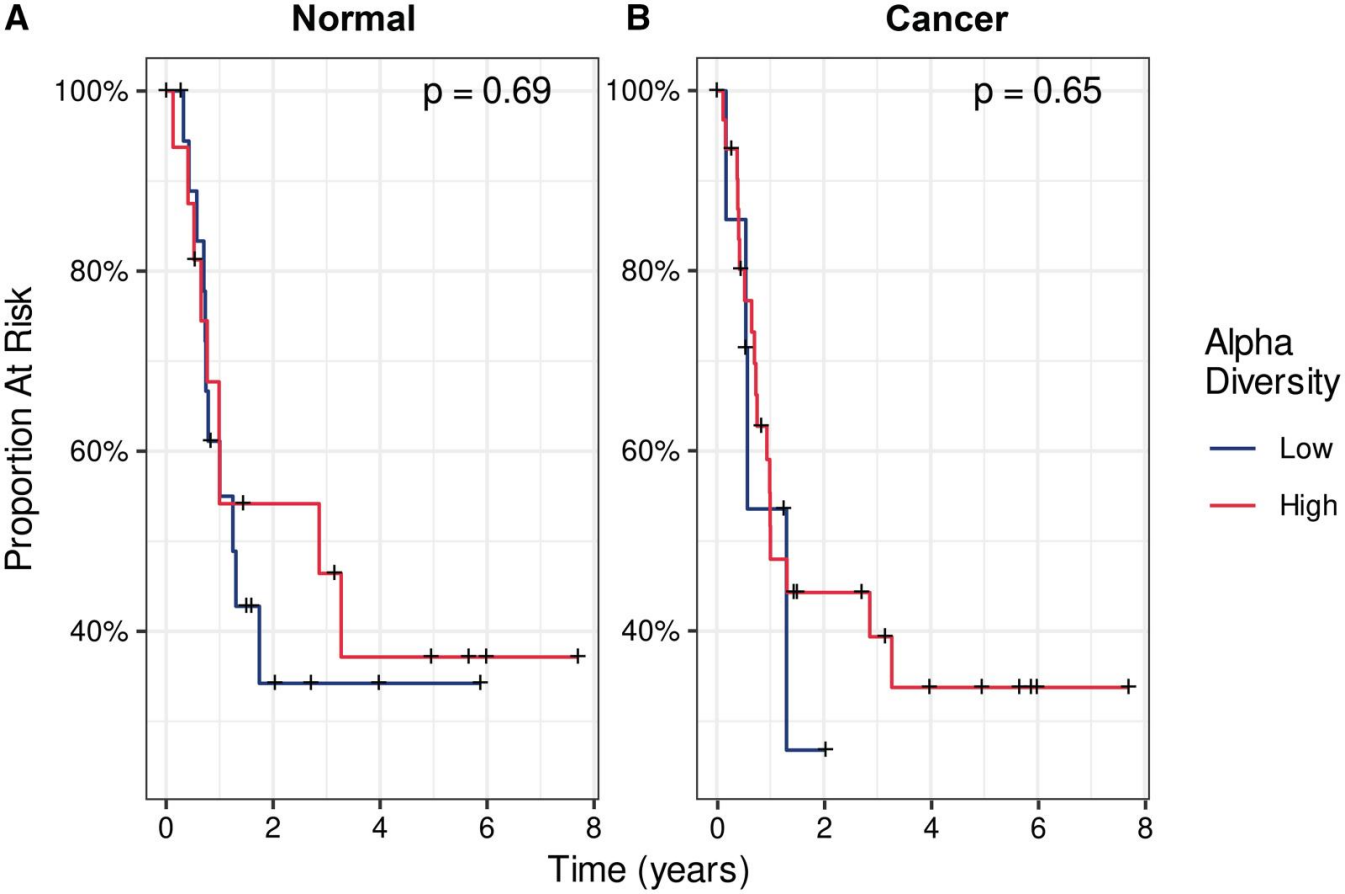
Kaplan-Meier analysis for patients with high alpha diversity (Shannon index ≥1) and low alpha diversity (Shannon index = 0) in **(A)** normal and **(B)** and tumor tissue.

Of the 72 patients in the cohort, 38 patients had follow-up of at least 2 years. Twenty-four patients experienced recurrence, metastasis, or death within 2 years, while 14 patients did not. Low alpha diversity was defined as a Shannon index of 0 and high diversity as a Shannon index of 1 or higher. Based on a log-rank test, no significant difference in overall survival was seen between OSCC patients with high or low alpha diversity, including in normal tissue (*P* = .69; Figure 4A) and tumor tissues (*P* = .65; Figure 4B).

## Discussion

This study maps the microbiome landscape of OSCC using WGS data from the largest cohort of young nonsmoking OSCC patients globally. Importantly, normal oral mucosa from healthy volunteers was used as the control. We extracted nonhuman bacterial reads using the taxonomic microbial classifier, MetaPhlAn v4.0.6 from the WGS data, a tool widely used for microbial profiling.^19^ We are the first to present differences in bacterial composition and abundance between normal mucosae from healthy volunteers and cancer patients and young and older OSCC patients. This includes significantly higher within-sample diversity in all tissue types from young OSCC patients, as compared with older OSCC patients, and a more anaerobic bacterial composition in young OSCC patients.

The paired tumor and matched normal sample analyses demonstrated significantly higher abundance in multiple different oral bacterial species in tumor compared with normal tissue. All bacterial species (Table 1) have been found to be enriched in OSCC in previous studies, including the well-characterized *Fusobacterium nucleatum.*^27^^,^^28^ More recently, the role of *F. nucleatum* in oral carcinogenesis has been revealed. *Fusobacterium nucleatum* can bind to and invade oral epithelial cells via adhesins, FadA and Fap2,^29^ and cause cellular proliferation via activation of Toll-like Receptor 4 and downstream pathways, including MYD88 signaling.^30^

A key finding is reduction in oral commensals like *S. mitis*, *H. haemolyticus*, and *C. acnes*. All 3 bacteria are constituents of the normal flora of the oral cavity^6^ and have been linked to immunomodulatory properties, including cytokine expression regulation.^28^^,^^31-36^ Our data demonstrate a reduction in these bacteria in the surrounding normal mucosa adjacent to OSCC. The impact of this reduction in oral commensals on the inflammatory milieu requires further investigation, directly examining the functional links between changes in bacterial composition and the immune microenvironment. Interestingly, Jain *et al*. report enrichment of *C. acnes* and *Malassezia restricta* using an ultradeep metagenomic approach in 20 patients.^37^

The paired sample analysis demonstrated a significant reduction in the abundance of 9 other anaerobic bacterial species in OSCC tissues from older patients as compared with younger patients. The most noteworthy include a combination of gram positive (*G. morbillorum*, *P. stomatis*, *F. alocis*) and gram negative (*A. segnis*, *P. endodontalis*) anaerobes. The anaerobes identified in our study have been implicated in endodontic infection (*G. morbillorum*),^38^ and periodontitis (*P. stomatis, F. alocis*, *and P. endodontalis*).^39^ The relationship between endodontic health in young patients and the development of OSCC warrants longitudinal studies in this patient population, particularly in relation to pregnancy and other environmental factors.

The presence of anaerobic bacteria *within* OSCC tissue, as opposed to merely on the surface, has been demonstrated previously; however, a difference in abundance according to age has not been evaluated.^40^^,^^41^ In young OSCC patients, we identified numerous other anaerobes beyond the well-characterized *F. nucleatum.* Proposed mechanisms for the presence of anaerobic bacteria *within* tumor tissue include inadequate blood supply enabling preferential growth in hypoxic tumor regions, as well as infiltration of anaerobes into the deep tumor from overlying tumor-associated biofilms.^40^ Such hypoxic conditions favor anaerobic bacteria—for example, *G. morbillorum* and *P. stomatis* can be aciduric and fermentative, with byproducts including lactic, acetic, and butyric acids.^42^ The findings of increased numbers and abundance of anaerobes in young patients with OSCC is hypothesis generating and the implications of a more acidic and hypoxic environment on carcinogenesis merits further investigation.^43^

The impact of age on within-sample microbial diversity has been explored in other cancers of the aerodigestive tract but not in OSCC. Ganly *et al*. compared the microbiome extracted from the mouthwashes of 42 nonsmoking OSCC cases to 45 matched nonsmoking controls, and did not identify a significant difference in within-sample diversity; the majority of the cohort was older than 50 years and the effect of age was not examined.^13^ A similar finding to ours has been noted in colorectal carcinoma,^44^ where younger patients were found to have higher within-sample diversity. However, the converse finding has been observed in gastric carcinoma, where within-sample diversity may be influenced by an age-related decline in immunity and mucosal atrophy.^45^ With specific reference to OSCC, the increased within-sample diversity in young patients implies that tumors from younger patients are more polymicrobial. Therefore, interventions such as screening for a specific microorganism, for example, *Helicobacter pylori* in gastric carcinoma, is unlikely to be clinically useful in young OSCC patients.

There are many methodological debates in the field of the cancer microbiome. We carefully followed the STORMS checklist for reporting in microbiome research, to ensure reproducibility. Robust methods were used including tissue collection ensuring cancer and normal tissues or various oral subsites are not combined. Studies that use oral swabs and oral rinses are likely to combine the bacterial flora from multiple oral subsites.^14^ These methods also examine only the tumor surface and not its substrate or microenvironment, yielding different bacterial communities and inconsistent beta diversity findings, as compared with testing of tumor tissue.^28^^,^^46^

Most studies use 16S rRNA sequencing in OSCC with its associated detection biases, due to its amplicon-based PCR amplification and different approaches to the sequencing of the 16S region.^13^^,^^14^^,^^13^^,^^14^ Metagenomic analysis represents an unbiased direct process; however, it has been plagued by recent controversies in cancer microbiome studies.^47^ We employed MetaPhlAn version 4.0.6, a metagenomic taxonomic profiler that uses unique species-specific gene markers, not found in other species (including humans).^19^ Taxonomic-based metagenomic approaches are preferred in the first instance, as there is a risk of under- and overestimation of species abundances via a sequence abundance profiler.^19^

Despite being the largest cohort of OSCC patients with WGS data and clinical outcomes, particularly in those <50 years of age, the main limitation of our study is its sample size of 72 individuals. The other limitation of our study is orthogonal validation. A future consideration for orthogonal validation may include spatial mapping of bacterial species within OSCC tissues, as demonstrated by Galeano Niño *et al*.^48^ Additionally, all our healthy controls were never smokers, whereas our patient cohort was a mixture of ever smokers and never smokers. Smoking has been shown to influence the oral microbiome,^49^ and therefore this should be controlled for in future studies. Another future challenge is to understand the temporal changes in the cancer microbiome, and the balance between bacteria that may be enabling cancer formation vs those that colonize the tumor and change its microbial composition.^36^

Overall, this study is the first to present differences in OSCC bacterial composition and abundance between young and older patients, with notable increases in the abundance of anaerobes in young patients. The clinical drivers of these compositional differences require further exploration, including any potential links to periodontal infection or pregnancy. We envisage that future studies of the OSCC microbiome will capture these dental health factors, which may provide further insight into the development of OSCC in younger patients.

## Supplementary Material

pkag022_Supplementary_Data

## Data Availability

The data underlying this article cannot be shared due to current restrictions under Australian and New South Wales legislation regarding deposition of genomic data in public repositories. As we are unable to share the WGS data, we have shared detailed sequencing coverage and quality statistics (Table S1), clinical data (Table S2), and bacterial profiles (species abundance) for each study participant (Table S3) to allow interrogation of our findings. Given that we are unable to deposit the WGS data in a public repository, interested individuals can contact the corresponding author to arrange a data transfer agreement between institutions.
