# Supplementary material for: The microbiome landscape of oral cancer in young patients
Source: JNCI Cancer Spectr. 2026 Apr 21;10(2):pkag022. doi: 10.1093/jncics/pkag022 (PMC13124277; doi:10.1093/jncics/pkag022)
Supplement: pkag022_Supplementary_Data [file pkag022_supplementary_data.zip › TablesS4andS5_clean.docx]

## Supplementary Materials

##### Table S4: Abundance of bacterial species in normal tissue from OSCC patients vs. control mucosa from healthy volunteers (adjusted p < 0.05, Benjamini-Hochberg). Negative log-fold changes indicate the species are higher in control mucosa.

|  | **log2 FC** | **SE** | **Stats** | **adj. p-value** |
| --- | --- | --- | --- | --- |
| *Streptococcus mitis* | -7.4 | 1.5 | -5 | 0.00027 |
| *Haemophilus haemolyticus* | -6.6 | 1.4 | -4.8 | 0.00035 |
| *Cutibacterium acnes* | -8.2 | 1.9 | -4.4 | 0.00091 |

##### Table S5: Abundance of bacterial species in paired samples - tumour vs. normal tissue (adjusted p < 0.05, Benjamini-Hochberg). Positive log-fold changes indicate the species are higher in cancer.

|  | **log2 FC** | **SE** | **Stats** | **adj. p-value** |
| --- | --- | --- | --- | --- |
| *Gemella morbillorum* | 3.5 | 0.76 | 4.6 | 0.0015 |
| *Aggregatibacter segnis* | 2.2 | 0.57 | 3.8 | 0.0094 |
| *Solobacterium moorei* | 2.6 | 0.72 | 3.7 | 0.01 |
| *Parvimonas micra* | 2.5 | 0.71 | 3.5 | 0.014 |
| *Capnocytophaga sputigena* | 3.6 | 1.1 | 3.3 | 0.022 |
| *Porphyromonas endodontalis* | 2.9 | 0.93 | 3.1 | 0.022 |
| *Peptostreptococcus stomatis* | 1.9 | 0.6 | 3.1 | 0.022 |
| *Prevotella nigrescens* | 3.5 | 1.1 | 3.1 | 0.023 |
| *Fusobacterium nucleatum* | 3.7 | 1.3 | 2.9 | 0.036 |
| *Dialister pneumosintes* | 2.3 | 0.81 | 2.8 | 0.038 |
| *Haemophilus haemolyticus* | 3.4 | 1.2 | 2.8 | 0.038 |
| *Filifactor alocis* | 2 | 0.72 | 2.7 | 0.038 |

##### 
