# Supplementary material for: The microbiome landscape of oral cancer in young patients
Source: JNCI Cancer Spectr. 2026 Apr 21;10(2):pkag022. doi: 10.1093/jncics/pkag022 (PMC13124277; doi:10.1093/jncics/pkag022)
Supplement: pkag022_Supplementary_Data [file pkag022_supplementary_data.zip › Supplementary Methods.docx]

**Supplementary Methods**

LinDA is linear modelling that is specifically tailored to analyse the compositional nature of microbiome data and considers that the abundance of each species is not independent of other species in a particular sample. Briefly, one linear model was fitted with cancer-normal pairing information and another model with only cancer samples. Not adjusting for lifestyle and environmental factors by analysing only tumour tissue samples has been shown to be detrimental to cancer microbiome analysis^19^ and a substantial fluctuation of coefficient statistics is observed between these two approaches (Supplementary figure 2).
